# Effect of Heat Stress on Seed Protein Composition and Ultrastructure of Protein Storage Vacuoles in the Cotyledonary Parenchyma Cells of Soybean Genotypes That Are Either Tolerant or Sensitive to Elevated Temperatures

**DOI:** 10.3390/ijms21134775

**Published:** 2020-07-05

**Authors:** Hari B. Krishnan, Won-Seok Kim, Nathan W. Oehrle, James R. Smith, Jason D. Gillman

**Affiliations:** 1 Plant Genetics Research Unit, USDA-Agricultural Research Service, Columbia, MO 65211, USA; nathan.oehrle@usda.gov (N.W.O.); jason.gillman@usda.gov (J.D.G.); 2 Plant Science Division, University of Missouri, Columbia, MO 65211, USA; wonseokk@missouri.edu; 3 Crop Genetics Research Unit, Stoneville, MS 38776, USA; rusty.smith@usda.gov

**Keywords:** soybean, elevated temperature stress, seed development, proteomics, protein storage vacuoles

## Abstract

High growth temperatures negatively affect soybean (*Glycine max* (L.) Merr) yields and seed quality. Soybean plants, heat stressed during seed development, produce seed that exhibit wrinkling, discoloration, poor seed germination, and have an increased potential for incidence of pathogen infection and an overall decrease in economic value. Soybean breeders have identified a heat stress tolerant exotic landrace genotype, which has been used in traditional hybridization to generate experimental genotypes, with improved seed yield and heat tolerance. Here, we have investigated the seed protein composition and ultrastructure of cotyledonary parenchyma cells of soybean genotypes that are either susceptible or tolerant to high growth temperatures. Biochemical analyses of seed proteins isolated from heat-tolerant and heat-sensitive genotypes produced under 28/22 °C (control), 36/24 °C (moderate), and 42/26 °C (extreme) day/night temperatures revealed that the accumulation in soybean seeds of lipoxygenase, the β-subunit of β-conglycinin, sucrose binding protein and Bowman-Birk protease inhibitor were negatively impacted by extreme heat stress in both genotypes, but these effects were less pronounced in the heat-tolerant genotype. Western blot analysis showed elevated accumulation of heat shock proteins (HSP70 and HSP17.6) in both lines in response to elevated temperatures during seed fill. Transmission electron microscopy showed that heat stress caused dramatic structural changes in the storage parenchyma cells. Extreme heat stress disrupted the structure and the membrane integrity of protein storage vacuoles, organelles that accumulate seed storage proteins. The detachment of the plasma membrane from the cell wall (plasmolysis) was commonly observed in the cells of the sensitive line. In contrast, these structural changes were less pronounced in the tolerant genotype, even under extreme heat stress, cells, for the most part, retained their structural integrity. The results of our study demonstrate the contrasting effects of heat stress on the seed protein composition and ultrastructural alterations that contribute to the tolerant genotype’s ability to tolerate high temperatures during seed development.

## 1. Introduction

Soybean was originally introduced into the United States as a forage crop [1], but has emerged as a preeminent oilseed crop, owing to the high quality and content of oil and protein in the seeds [2]. Soybean global production in 2018 was estimated at 13.3 billion bushels, with the United States being the largest producer, accounting for 34.3% of total production (http://soystats.com/ accessed 14 April 2020). The Delta region of Mississippi, in the Midsouth soybean growing region, frequently experiences reduced precipitation late in the growing season [3]. Producing soybeans using photoperiod-responsive lines typical of the latitude (MG5-7, planted in May/June and harvested in October) without irrigation has been associated with decreased seed yield. To remedy these issues, an agronomic shift was made to plant earlier-maturing varieties (MG3-4) as early as possible (typically April), which would mature before the majority of drought stress could negatively impact seed development, with harvest occurring in late August and September. This practice has become known as the early soybean production system (ESPS) [1], and has been shown to increase on-farm seed yield and revenue under both irrigated and non-irrigated conditions [3].

Seed development in plants is sensitive to elevated temperatures [4,5]. In soybean, the optimum reproductive temperature is between 22–24 °C [6]. Although widely adopted in the Delta region of Mississippi, the practice of ESPS also frequently results in impaired seed quality and economic loss (dockage) at seed elevators [7,8], due to the presence of unacceptable levels of damaged seed (e.g., weathered, heat damaged, green, and mold), related to seed maturation during periods of high heat and humidity. Even in the absence of obvious seed damage, seeds are often impaired physiologically in their ability to germinate and/or emerge successfully when produced under consistently elevated temperatures using typical soybean lines [8].

Smith et al. performed a germplasm screen to identify soybean landraces that express tolerance to high temperatures in the ESPS [8]. As part of this study, the ancestors of modern high- yielding cultivars in North American were examined, and found to largely lack tolerance to elevated temperatures. However, an exotic landrace from China, PI587982A, was found to have tolerance to elevated temperatures and maintain near-perfect seed quality (>90% germination, virtual absence of *Phomopsis longicolla* infection) under conditions that severely impaired seed quality of most genotypes tested [8]. PI587982A has been used as a donor parent in multiple efforts to introgress tolerance alleles into elite germplasm, as well as mechanistic studies on the physiology and genetics of the tolerance trait. Gene expression responses for seed of PI587982A during the course of germination have been recently examined via RNASeq [9] and a range of antioxidant and temperature responsive genes were found to be expressed at higher levels in PI587982A, as compared to control seed.

DS25-1 (04025-1-1-4-1-1) is the first improved elevated temperature-tolerant public line released [10] and has been examined using a range of different methods in an attempt to identify and characterize tolerance mechanisms. In a recent metabolomics study [11], DS25-1 seeds were found to contain significantly elevated levels of flavonoids and tocopherols. The levels of these compounds were elevated under both optimal conditions and when seeds were produced under elevated temperatures (>30 °C). Germination of DS25-1 seed was unaffected by elevated temperatures, compared with a 50% reduction in germination response for seed from a line typical of cultivars grown in the Midsouth region [9,11]. Recently, a lipidomic-focused study found that leaves of DS25-1 grown under elevated temperatures have decreased polyunsaturated (linolenic acid, 18:3) lipid content, as well as a decline in the expression of certain key genes (*FAD3A/B*) encoding fatty acid desaturation enzymes [12].

The two major storage constituents of soybean seed are protein and oil. Soybean seeds of typical lines accumulate 36–40% protein and ~20% oil [2] and these two important components are stored in specialized structures in soybean seed; protein storage vacuoles (PSV) accumulate seed storage proteins, whereas oil is stored within lipid bodies [13,14,15]. While several studies have been conducted on the effect of elevated temperature on the overall oil and protein content of seed [12,16,17], only limited information is available on the effect of heat stress on the ultrastructure of the storage organs. Additionally, previous studies that employed omic approaches were focused on global changes in the metabolite profile and gene expression [9,11,12,17]. Since protein is the most important component of soybean seed, a detailed understanding of the effect of heat stress on the accumulation of seed proteins is vital to mitigate the adverse effect of heat stress. This study is aimed at elucidating the effect of heat stress specifically on soybean seed protein composition and to investigate changes in the ultrastructure of cotyledonary parenchyma cells, if any, between heat stress-tolerant and sensitive soybean seeds.

## 2. Results

### 2.1. Accumulation of Lipoxygenase, the β-Subunit of β-Conglycinin and Bowman-Birk Protease Inhibitor in Soybean Seeds Is Negatively Impacted by Heat Stress

**Figure 1:**
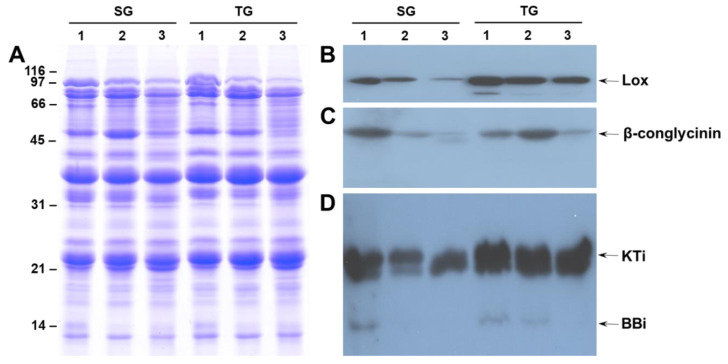
One-dimensional sodium dodecyl sulfate–polyacrylamide gel electrophoresis (SDS-PAGE) analysis of soybean total seed proteins (Panel **A**). Total seed proteins extracted from heat stress sensitive soybean genotype DT97-4290 (SG) and heat stress tolerant soybean genotype DS25-1 (TG) genotypes grown at 28/22 °C (lane 1) or 36/24 °C (lane 2) and 42/26 °C (lane 3) day/night temperatures were analyzed by 13.5% SDS-PAGE. Resolved proteins were detected by staining the gel with Coomassie Blue (Panel **A**) or transferred to nitrocellulose membrane and probed with antibodies raised against lipoxygenase1 (Panel **B**), the β-subunit of β-conglycinin (Panel **C**) and Bowman-Birk inhibitor (Panel **D**). The molecular weight markers whose sizes in kilodaltons are shown on the left side of the figure. The identity and approximate position of major seed storage proteins is indicated by arrows.

To study the effect of elevated growing temperature on seed protein composition we first examined the total protein profile by sodium dodecyl sulfate–polyacrylamide gel electrophoresis (SDS-PAGE) analysis. Figure 1 depicts the protein profile of seeds from heat-tolerant and heat-susceptible genotypes produced under 28/22 °C, 36/24 °C, and 42/26 °C day/night temperatures. Both the soybean genotype DS25-1 (TG) and soybean genotype DT97-4290 (SG) had comparable protein profiles, and failed to identify presence or absence of proteins unique to either genotype. The seed protein profile of seeds produced under 28/22 °C (control), and 36/24 °C day/night temperatures of both TG and SG were very similar to each other (Figure 1A). However, seeds produced under 42/26 °C day/night temperatures had a noticeable decline in the accumulation of a small number of proteins, with sizes of 94 kDa, 52 kDa, and 14 kDa (Figure 1A).

Based on the molecular weight and the results from our previous studies, we suspected that the 94 kDa, 52 kDa and 14 kDa proteins were lipoxygenase, the β-subunit of β-conglycinin and Bowman-Birk protease inhibitor, respectively. In order to confirm this hypothesis, we performed western blot analysis with polyclonal antibodies that specifically recognize these proteins. Antibodies raised against soybean lipoxygenase1 specifically reacted against a 94 kDa protein (Figure 1B). A strong signal was detected in the seeds produced under 28/22 °C for both the SG and TG genotypes, but only a week signal was detected in SG at higher temperatures, indicating low accumulation of the lipoxygenase protein. Antibodies raised against the soybean β-subunit of β-conglycinin reacted strongly against the 52 kDa protein from the seeds produced at 28/22 °C, whereas only a faint reaction was detected at higher temperatures (Figure 1C). In contrast, in the TG, the strongest reaction was detected in the seeds produced at 36/24 °C, indicating moderate heat stress actually prompted the accumulation of higher levels of β-subunit of β-conglycinin. In that both SG and TG seed development under extreme heat stress, a drastic reduction in the accumulation of the β-subunit of β-conglycinin was observed (Figure 1C). Similarly, antibodies raised against the soybean chymotrypsin inhibitor—the Bowman-Birk inhibitor (BBI)—reacted strongly against the 21 kDa trypsin inhibitor and the 14 kDa BBI proteins (Figure 1D). The trend in accumulation in SG and TG seed for BBI was very similar to that noted for the β-subunit of β-conglycinin (Figure 1C). These results indicate that the heat-tolerant genotype’s seed proteome was less impacted by seed growth under elevated temperatures compared to the heat-sensitive genotype.

### 2.2. 2D-Gel Electrophoresis Reveals the Negative Effect of Heat Stress on Seed Storage Protein Accumulation

**Figure 2:**
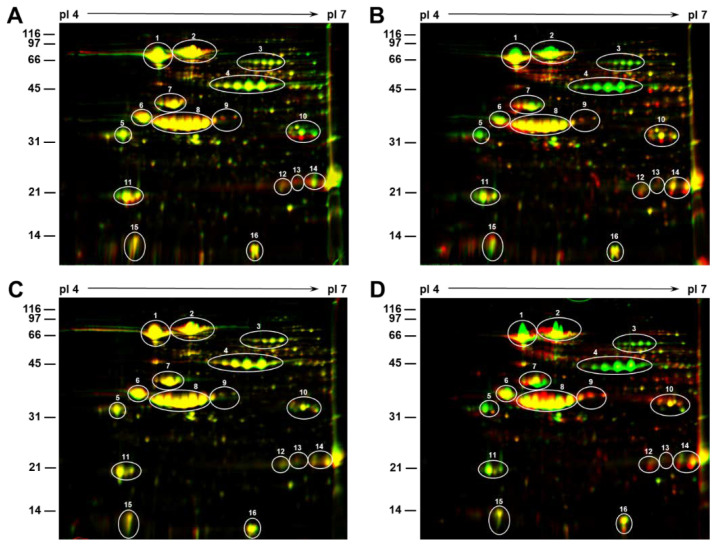
Effect of growing temperature on the seed proteins isolated from tolerant (TG) and heat stress sensitive (SG) genotypes. Seed proteins (300 μg) were first separated by isoelectric focusing on pH 4–7 strips followed by separation by SDS-PAGE on 16% gels. The gels were stained with colloidal Coomassie blue G-250. Gels were scanned and the resulting images were assigned two different colors (green = 28/22 °C and red = 36/24 °C and 42/26 °C), in order to visualize the differences between the two. For observing heat stress induced protein changes two identically separated 2D gels of soybean seed proteins grown at different temperatures were overlaid using Delta2D software. (**A**) Overlay between SG grown at 28/22 °C and 36/24 °C; (**B**) overlay between SG grown at 28/22 °C and 42/26 °C; (**C**) overlay between TG grown at 28/22 °C and 36/24 °C; and (**D**) overlay between TG grown at 28/22 °C and 42/26 °C. The superimposed color images generated by Delta2D software facilitates the identification of differences between the two treatments where yellow demonstrates similar protein quantities in both growing temperatures. The green color demonstrates absence of that particular protein species in the seeds grown at higher temperature and the presence of the red color demonstrates the accumulation of that particular protein species in the seeds grown at higher temperature. Spots 1, 2, and 4 represent the 7S -conglycinin subunits and spots 6–10, 12–14, and 16 represents the different glycinin subunits. Spots 3, 5, 11, and 15 are the sucrose binding proteins, p34 thiol protease, the Kunitz trypsin inhibitor (KTI) and the Bowman-Birk inhibitor (BBI), respectively.

The resolution power of 1D gels is limited, so we performed high-resolution 2D-gel electrophoresis to detect heat stress-induced seed protein alterations (Figure 2 and Figure S1). First, we compared the protein profile of SG seeds produced under 28/22 °C (control) and 36/24 °C day/night temperatures. For this purpose, seed proteins from these two treatments were resolved on two gels under identical conditions. Images are assigned two different colors (green = 36/24 °C and red = 28/22 °C) and are superimposed using Delta2D software (Figure 2A). Protein spots colored yellow indicate similar protein quantities in both treatments, the green color demonstrates lower levels of a protein species in the 36/24 °C treatment, and the red color indicates elevation of a particular protein species in the 36/24 °C treatment. Most of the 2D resolved protein spots in this comparison are yellow, indicating that shifting the growing temperature from 28/22 °C to 36/24 °C does not cause major alterations in the most abundant seed protein profile (Figure 2A,C). However, several green protein spots representing less abundant seed proteins were also present when SG seeds were grown at 36/24 °C (Figure 2A). In contrast, a comparison of the protein profile of SG seeds produced under 28/22 °C (control) and 42/26 °C day/night temperatures revealed striking differences (Figure 2B). The accumulation of several abundant seed protein spots (green spots) were drastically reduced in the seeds grown at 42/26 °C (Figure 2B). Based on the molecular weight, isoelectric point and mass spectrometric analysis [18,19,20] these protein spots have been identified as sucrose binding proteins, β-subunit of β-conglycinin and P34, a probable thiol protease.

Next, we compared the protein profile of TG seeds produced under 28/22 °C (control) and 36/24 °C (Figure 2C) and 28/22 °C and 42/26 °C (Figure 2D) day/night temperatures. Shifting the growth temperature from 28/22 °C to 36/24 °C had very little effect on the seed protein profile of the TG. Unlike the SG, where heat stress induced changes in a few minor seed proteins, the TG revealed no obvious changes as evidenced by the predominance of the yellow protein spots (Figure 2C). However, a comparison of protein profile of TG seeds produced under 28/22 °C and 42/26 °C revealed a drastic reduction (Figure 2D) in the accumulation of the same protein spots that observed in the SG (Figure 2B), indicating that 42/26 °C treatment has a profound effect on seed protein accumulation in both the SG and TG.

### 2.3. Effect of Heat Stress on the Accumulation of HSP70, HSP17.6 and BiP in Seeds of Heat-Tolerant and Heat-Sensitive Genotypes

**Figure 3:**
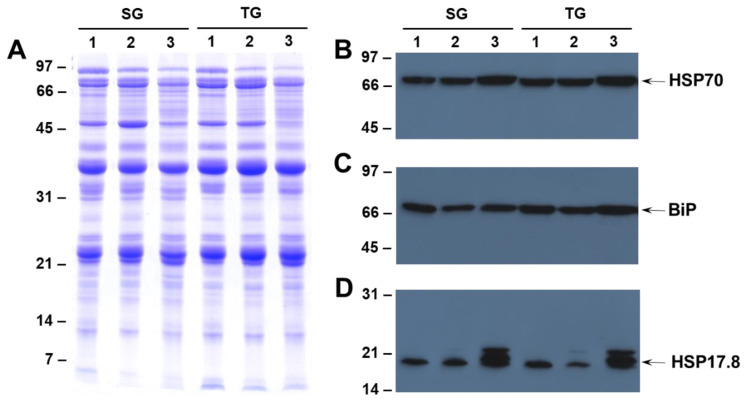
Immunological detection of heat shock and immunoglobulin heavy chain-binding proteins in tolerant (TG) and heat stress sensitive (SG) genotypes. Seed proteins were extracted from seeds of heat tolerant (TG) and heat-sensitive (SG) genotypes grown at different temperatures and separated by SDS-PAGE (**A**). Resolved proteins were electrophoretically transferred to nitrocellulose membranes and probed with (**B**) heat shock protein70 (HSP70); (**C**) immunoglobulin heavy chain-binding protein (BiP); and (**D**) heat shock protein17.6 (HSP17.6); specific antibodies. Immunoreactive proteins were identified using anti-rabbit IgG-horseradish peroxidase conjugate followed by chemiluminescent detection. The numbers on the left side of the figure indicate the sizes of protein standards in kilodaltons.

Earlier studies that focused on transcriptome profiling indicated that heat shock proteins may play an important role in improving the tolerance of soybean subjected to heat stress [21,22]. To examine if different growing temperatures had any effect on heat shock protein (HSP) accumulation, we conducted western blot analysis using antibodies specific for HSP70 and HSP17.6. The accumulation of HSP70 in SG and TG seeds produced at different growing temperatures displayed a gradual increase that coincided with increased temperature (Figure 3B). Similarly, the accumulation of the HSP17.6 was much higher in both the SG and TG seeds produced at 42/26 °C day/night temperatures (Figure 3D). In addition to the HSP17.6, the antibody also reacted strongly against the 22 kDa protein (Figure 3D). Interestingly, the accumulation of this protein, was not seen in the seeds produced at 28/22 °C, but trace amounts of accumulation were detected in the seeds produced at 36/24 °C (Figure 3D). We also examined the accumulation of immunoglobulin heavy chain-binding protein (BiP), a member of HSP70 family and a resident endoplasmic reticulum (ER) protein that plays a major role in chaperoning newly synthesized proteins and targeting misfolded proteins for degradation by the proteasome [23]. Antibodies raised against maize immunoglobulin heavy chain-binding protein (BiP) specifically reacted against the 66 kDa protein from soybean seeds (Figure 3C). However, unlike HSP70 and HSP17.6 the accumulation of BiP was not noticeably affected by heat stress in either the SG and TG seeds (Figure 3C).

### 2.4. Ultrastructure Examination of Soybean Cotyledonary Cells Show That Heat-Tolerant Genotype Maintains Cellular Integrity Superior to the Heat-Sensitive Genotype at Elevated Temperatures

**Figure 4:**
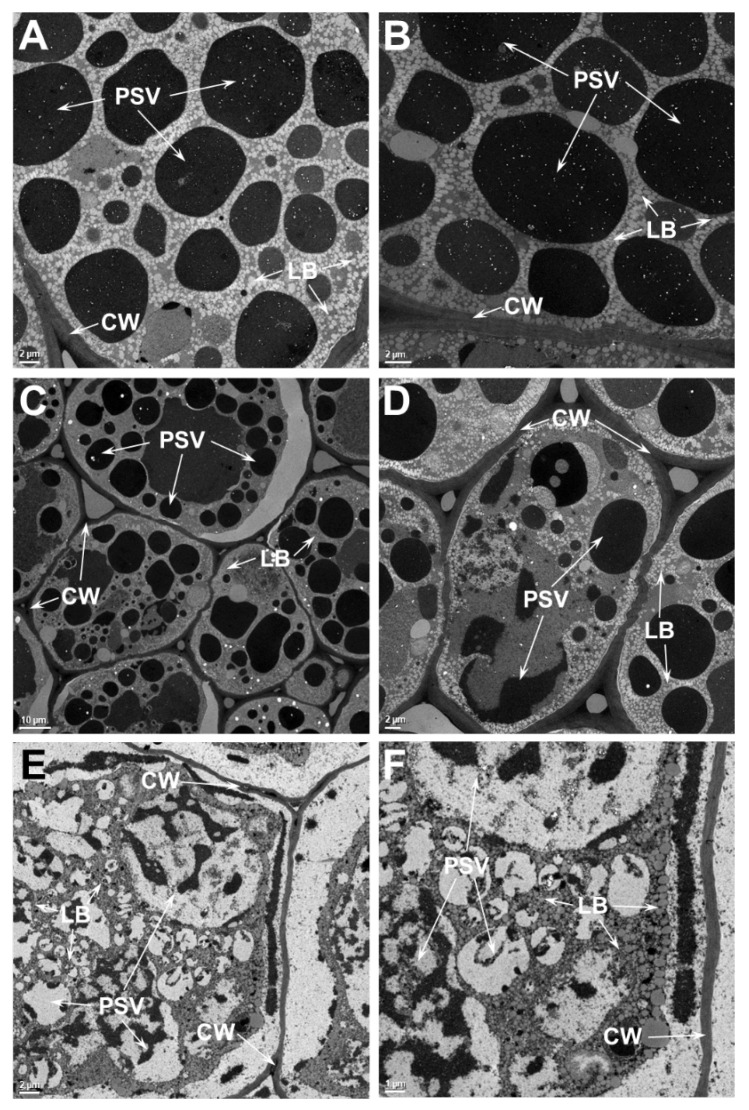
Transmission electron micrographs of heat stress sensitive (SG) soybean cotyledon grown at different temperatures. Low and high magnification micrographs of soybean cotyledons that were grown at 28/22 °C (**A**,**B**) or 36/24 °C (**C**,**D**) and 42/26 °C (**E**,**F**) day/night temperatures. Numerous protein storage vacuoles and lipid bodies are observed in these cells. Note the distortion of protein storage vacuoles are apparent even at 36/24 °C (**C**,**D**) and the complete loss of structural integrity in the cotyledonary cells of sensitive soybean genotype grown at 42/26 °C (**E**,**F**). PSV, protein storage vacuoles; LB, lipid bodies; CW, cell wall.

The two main storage reserves of soybean, protein and oil, accumulate in the seed cotyledonary parenchyma cells [24]. In these cells, the storage proteins are stored in specialized vacuoles called protein storage vacuoles (PSV) and the oil is stored in lipid bodies (LB). In order to evaluate the effect of heat stress on the ultrastructure of seed cotyledonary parenchyma cells, we examined the thin sections of seed tissue by transmission electron microscopy (Figure 4). An examination of the parenchyma cells of the SG that was grown at 28/22 °C reveals that the cells are filled with numerous osmophilic dark staining PSV of various sizes (1.5 μm–14.8 μm). Dispersed within these PSV, small phytate inclusions were observed (Figure 4A,B). Occasionally, the presence of a nucleus was also observed. Other organelles, including rough ER and Golgi apparatus, were not observed in these cells. Observation of parenchyma cells of the SG that was grown at 36/24 °C presented almost similar anatomy as that was observed in cotyledons grown at 28/22 °C (Figure 4C,D). Though these cells contained numerous PSV, the size and shape of these storage organelles were somewhat distorted. Often, these PSVs were larger (greater than 18.5 μm), and exhibited two regions that showed different staining pattern (Figure 4D). Often, in these cells the plasma membrane was pulled away from the cell wall (Figure 4C). Additionally, some of the LB were much larger than those observed in the cells from the 28/22 °C treatment. The appearance of parenchyma cells from the 42/26 °C treatment was strikingly different from the other treatments (Figure 4E,F). No discrete PSV were observed. Instead, several vacuoles containing dark staining ramifying threads were found (Figure 4E,F). Similar dark-staining threads were also observed outside these vacuoles as tubular structures close to the cell walls (Figure 4E,F).

**Figure 5:**
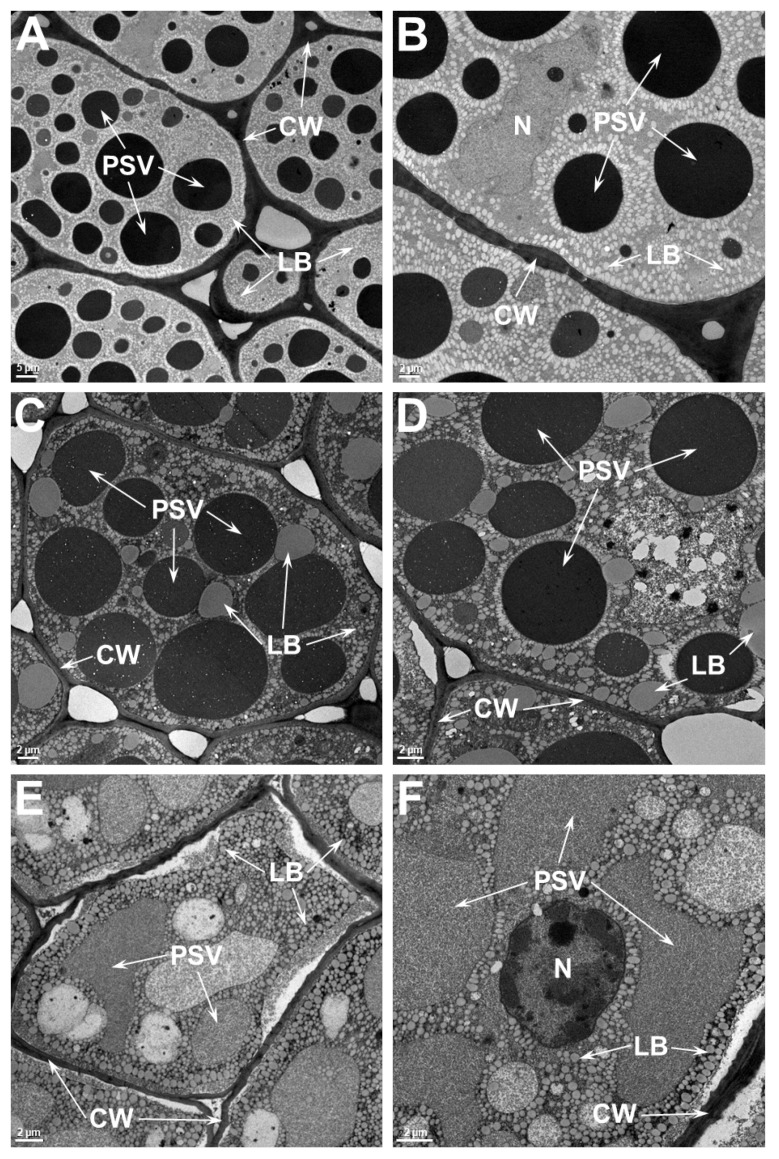
Transmission electron micrographs of tolerant (TG) soybean cotyledon grown at different temperatures. Low and high magnification micrographs of soybean cotyledons that were grown at 28/22 °C (**A**,**B**) or 36/24 °C (**C**,**D**) and 42/26 °C (**E**,**F**) day/night temperatures. Numerous protein storage vacuoles and lipid bodies are observed in these cells. Note the distorted appearance and size of protein storage vacuoles in the cotyledonary cells of tolerant soybean genotype grown at 42/26 °C. PSV, protein storage vacuoles; LB, lipid bodies; CW, cell wall; N, nucleus.

An examination of the parenchyma cells of the TG that was grown at 28/22 °C (Figure 5A,B) reveals almost the same appearance as that was observed in the SG. The parenchyma cells were filled with several PSV of various sizes (1.2 μm–18 μm), dispersed among small spherical lipid bodies. The parenchyma cells from the 36/24 °C treatment (Figure 5C,D) appeared very similar to those of the 28/22 °C treatment (Figure 5A,B), with no obvious aberrations due to heat stress. In contrast to the situation seen in the SG (Figure 4A,B), the plasma membrane in TG cells were not pulled away from the cell wall (Figure 5C,D). However, this situation was only noticeable in cells at the 42/26 °C treatment (Figure 5E,F). More importantly, these cells still contained large PSVs with light-staining material. Some of the PSV appeared granular suggesting incomplete filling with storage proteins (Figure 5E,F). The appearance of LBs was not noticeably different from than observed in the cells from the other temperature treatments.

**Figure 6:**
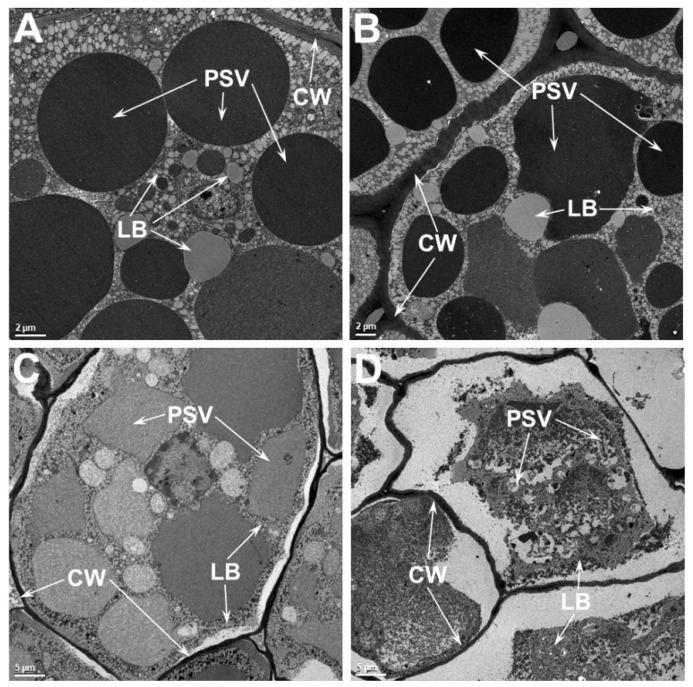
Transmission electron micrographs of tolerant (**A**,**C**) and sensitive (**B**,**D**) soybean genotypes that were grown at different temperatures. Micrographs of soybean cotyledons that were grown at 36/24 °C (**A**,**B**) or 42/26 °C (**C**,**D**) day/night temperatures. Numerous protein storage vacuoles and lipid bodies are observed in these cells. Note the distorted appearance and size of protein storage vacuoles in the cotyledonary cells of the tolerant soybean genotype and the complete loss of internal structural organization in the sensitive genotype grown at 42/26 °C (**C**,**D**), respectively. PSV, protein storage vacuoles; LB, lipid bodies; CW, cell wall.

A side by side ultrastructural comparison between SG and TG seeds indicated no obvious differences between the two when grown at 28/22 °C. However, at higher temperatures, clear differences in the organelle integrity are observed between these two genotypes. The structures of the PSV and LB in the TG parenchyma cells are well preserved when the seeds were grown at 36/24 °C, while the seeds of the SG show indications of loss of membrane integrity (Figure 6A). In SG cells, the plasma membrane is often seen pulling away from the cell wall (Figure 6B). The PSVs, which are mostly spherical in the TG, are often convoluted and their contents reveal less intense staining in SG (Figure 6B). The difference between TG and SG seed ultrastructure is pronounced when they were grown at 42/26 °C (Figure 6C,D). Even at this high temperature, above the reported lethal temperature for soybean seeds [6] the structure of cotyledonary cells is still largely preserved in the TG (Figure 6C). Even though the PSV have lost their typical spherical shape, a distinct intact membrane around them is still observable. The other obvious adverse effect of heat stress is reflected by the plasma membrane pulling away from the cell walls. In contrast, a dramatically different response is seen in the cotyledonary cells of the SG (Figure 6D). The PSV in these cells have completely broken down. The contents of PSV appear to be intermingled with LB and dispersed throughout the cell cytoplasm. No intact plasma membrane is noticeable, and the cell cytoplasm is completely pulled away from cell walls, presumably due to plasmolysis (Figure 6D).

## 3. Discussion

Previous studies have established that heat stress leads to reduced seed weight, seed quality, germination and vigor, increased seed wrinkling, impermeable seed coat, and sometimes a higher incidence of pathogen infection [3,8,11,25]. It has been reported that seed protein and N content increase with increasing growing temperature and that there is a negative correlation between protein and oil across a range of growing temperatures [26,27,28,29]. Earlier, we reported similar effects of heat stress on the seed quality traits of heat stress-tolerant and sensitive soybean genotypes grown under 28/22 °C, 36/24 °C, and 42/26 °C day/night temperatures [11]. Overall, the percentage seed nitrogen (protein) concentration in the SG was significantly increased with an increasing growing temperature, while in TG, it remained almost unchanged at 28/22 °C and 36/24 °C, and decreased marginally at 42/26 °C day/night temperatures [11]. In this study, we conducted additional studies focused on the effect of heat stress on soybean seed protein composition and the ultrastructure of cotyledonary parenchyma cells. SDS-PAGE analysis of total seed proteins based on equal amounts of seed powder revealed that heat stress produced a negative effect on the accumulation of few seed proteins in both the TG and SG lines (Figure 1). Our immunological studies demonstrated that heat stress profoundly decreased the accumulation of lipoxygenase, the β-subunit of β-conglycinin and the Bowman-Birk protease inhibitor. Our proteomic analysis also identified few other proteins whose accumulation was affected by high temperature. The most dramatic effect of heat stress (42/26 °C) was the loss of accumulation of the β-subunit of β-conglycinin, sucrose binding protein and p34 thiol protease (Figure 2).

A previous proteomic analysis of soybean seeds produced at 27/18 °C and 37/30 °C identified several proteins whose accumulation was changed by high temperature [16]. A total of 20 proteins spots were identified by mass spectrometry, out of which fourteen spots were identified as different subunits of glycinin and β-conglycinin. Even though heat stress mostly increased the accumulation of different subunits of 11S and 7S globulins, it also lowered the accumulation of other subunits of glycinin and β-conglycinin. Our results are consistent with this earlier study. However, the previous study reported an increase in Bowman–Birk type proteinase inhibitor D [30] in seeds produced under high temperature, relative to seeds from the control temperature [16]. However, in our current study, we found that when the growing temperature was increased to 42/26 °C, the accumulation of Bowman-Birk protease inhibitor was inhibited. This observation was validated by western blot analysis using antibodies that were specific to soybean Bowman-Birk protease inhibitors. One possible explanation for lower accumulation of BBI under extreme heat stress may be related to increased N content in seeds produced at higher growing temperatures. Previously, we have shown that nitrogen had a negative influence on the expression of the BBI genes in soybean seed [31].

Lipoxygenase (LOX), a group of non-heme metal-containing dioxygenases, are particularly abundant in grain legume seeds [32]. Soybean contain at least three isoforms of the LOX enzyme, which can be differentiated based on their substrate preference, optimal pH, product formation, and stability. The precise function of LOXs in soybean seeds are not well understood. Various physiological functions have been proposed including a role in plant growth and development, senescence, and wound responses [33], pest and disease resistance [34,35], and the temporary storage of N in vegetative tissue [36]. Hexanal, derived from the LOX-catalyzed hydroperoxidation of linoleic acid, has been shown to be primarily responsible for flavors perceived as grassy or beany [37,38,39,40]. However, studies employing lipoxygenase-free soybean mutant line [41] have shown that LOXs may not have major role in generating grassy or beany flavors. Our results reveal that heat stress differentially affects the accumulation of LOX1 in SG and TG lines (Figure 1B). Even under extreme heat stress, the accumulation of LOX1 in the TG line is not severely affected, when compared to the SG line. Our observation thus raises a question regarding the role of LOX1 in heat stress-induced seed deterioration. If LOXs are major contributors to lipid peroxidation and free radical generation, then seeds accumulating higher amounts of LOX would experience more cellular damage. However, our ultrastructural studies show that the membrane integrity is much better preserved in the TG than in the SG line, indicating that LOX1 may not play a direct role in maintaining seed vigor and viability. In fact, it has been shown using LOX mutants that the loss of one or two of the three LOX isozymes had no effect on soybean seed deterioration [42].

Heat shock proteins (HSPs) are a group of proteins produced by most eukaryotic cells, in response to supra-optimal high temperature [43]. These proteins are implicated in protecting plants against abiotic stresses. They act as molecular chaperones assisting in protein folding or unfolding for intracellular distribution, assembly, and degradation to maintain protein homeostasis [44]. The HSPs are classified into five major families based on their molecular weights: small heat shock protein (smHSP), chaperonin family (HSP60), 70-HSP70, HSP90, and HSP100 [45]. The HSP70 family member, BiP, plays a major role in chaperoning newly synthesized proteins, maintaining the permeability barrier of the ER during protein translocation, and targeting misfolded proteins for eventual degradation by proteasome [46]. Additionally, a role for HSPs in improving seed vigor also been suggested [46]. A previous study reported an increased accumulation of HSP22 in seeds developed under the high temperature treatment (37/30 °C), relative to seeds from the control temperature (27/18 °C) regime [16]. In contrast, we observed an increased accumulation of HSP70 and HSP17.6 only when the seeds were produced under extreme heat stress (42/26 °C). The accumulation of these HSPs was not noticeably different in seeds produced under the moderate heat stress (36/24 °C), compared to seeds from the control temperature (28/22 °C). Interestingly, both the SG and TG lines accumulated similar amounts of HSPs when subjected to heat stress (Figure 3). Thus, it is not clear if the accumulation of HSP70 and HSP17.6 had any direct role in the ability of the TG line to better cope with high temperature damage. However, our observation does not rule out the possibility that other members of HSPs may be involved in providing protection against heat stress. It is notable that HSPs are encoded by multigene families, and the W82 soybean genome contains at least 61 HSP70 encoding genes, which are differentially expressed by heat stress in a tissue-specific manner [22].

Earlier studies have shown high temperature stress affects cell ultrastructure, causes progressive disintegration of the nucleolus and the assembly of cytoplasmic heat shock granules, and damages the cellular membranes [47,48,49]. High temperature stress was shown to damage the plasma membrane, chloroplast membrane, and thylakoid membranes in soybean leaves [50]. Thus, damage to cellular membranes is a hallmark of heat stress response. In this regard, it should be pointed out that seeds produced at high temperature exhibit decreased seed vigor, which is caused by the loss of integrity of cellular membranes. Seeds produced at high temperatures reveal increased electrical conductivity, indicating greater electrolyte leakage presumably due to damaged cellular membranes [16]. The fluidity and permeability properties of biological membranes, which are composed of lipids and proteins, can be effectively altered by elevated temperatures. Heat stress can lead to microdomain remodeling of membranes and activate downstream signaling pathways [51]. The plasma membrane (PM), and membranous organelles play a major role in determining the cell’s fate during plant adaptation to heat stress. Lipid peroxidation, which leads to the generation of chemically reactive products, contributes to the loss of cellular functions.

Our ultrastructural analysis of soybean seeds revealed a loss of integrity of cellular membranes in the seeds produced at moderate and extreme temperatures (Figure 6). A comparative examination of the transmission electron micrographs of seed produced under heat stress clearly demonstrates that the TG seed maintain superior structural integrity of cellular membranes as compared SG seeds. So, the question remains: what enables the TG to cope with the deleterious effect of heat stress? Answers to this question can be gleamed from our recent study on the impact of high temperatures on global seed metabolites: we identified several antioxidant metabolites (including tocopherols, flavonoids, phenylpropanoids, and ascorbate precursors) were enriched in seeds of the TG when compared to the SG [11]. Ferulic acid, an antioxidant and important structural component of the plant cell wall [52], was found to be higher (9-fold) in TG seed as compared to SG seed. Similarly, the levels of tocopherols, potent lipophilic antioxidant molecules, were also reported to be elevated in the seeds of the TG line. Tocopherols are known to stabilize the plasma membranes during heat stress, implicated in scavenging reactive oxygen species (ROS), and can prevent membrane damage and lipid peroxidation [53]. Elevated levels of these antioxidant metabolites in the TG line may play a protective role in minimizing the deleterious effects of high growth temperature. An earlier systematic study, which reported the effects of growth temperature and carbon dioxide enrichment on soybean seed components at different stages of development, found the levels of key metabolites were altered during the initial stages of seed development [29]. However, our study was limited to a single timepoint (mature seed) and, as such, did not investigate potential temporal differences between the TG and SG in accumulation of these antioxidant metabolites. One hypothetical possibility is that the TG could have earlier and more robust responses to heat stress compared to SG. Such an experimental is not a trivial undertaking, however, and would require measuring multiple antioxidant metabolite levels, as well as ultrastructural comparisons, across multiple seed developmental stages (e.g., cell division, grain filling, and desiccation).

## 4. Materials and Methods

### 4.1. Plant Materials

This study utilized two soybean lines: (1) DS25-1, a line which was publicly released [10], and has been demonstrated to have high tolerance [10,11,12] to the deleterious impacts of elevated temperatures during seed fill; (2) and a high-yielding, but heat-sensitive line ‘DT97-4290′ [54]. The heat tolerant and heat-sensitive genotypes used in this study will be referred from this point onwards as TG and SG, respectively. Twenty-four (12 TG and 12 SG seeds) were individually planted in 2-gallon pots containing PRO-MIX medium (Premier Horticulture, Quebec City, QC, Canada). For each treatment four replications were included. Plants were grown for their entire life cycle in Conviron E15 growth chambers. Plants were fertilized with Osmocote Plus (Scotts, Marysville, OH, USA) every 15 days, and watered as needed. Day night cycle was 14 h day/10 h night. Plants were initially started at 28/22 °C day-night temperatures and when flowering had initiated were either (1) maintained at a control temperature (28/22 °C day-night), or (2) moved to a moderate heat stress regime of 36/24 °C (day/night), or a high-temperature (extreme) stress regime of 42/26 °C (day/night). These temperature regimes were maintained through pod fill. After the seeds matured, they were harvested and stored at ambient (−25 °C) laboratory conditions.

### 4.2. 1D and 2D Electrophoresis

The protocol used to isolate seed proteins and resolving them by 1D and 2D gel electrophoresis followed by image acquisition and analysis have been described previously [19,20].

### 4.3. Immunoblot Analysis

Immunoblot analysis was performed as described earlier [31]. The following antibodies were used in this study: soybean lipoxygenaase1 [55], soybean BBI [56], and soybean-conglycinin-subunit [57]. HSP17.6 antibodies were purchased from AgriSera (AS07 254, Vännäs, Sweden), whereas HSP70 and BiP antibodies were obtained from Drs. Jill Winter and Rebecca Boston, respectively.

### 4.4. Transmission Electron Microscopy

Mature dry seeds harvested from both genotypes grown at ether 28/22 °C (control), 36/24 °C (moderate), or 42/26 °C (extreme) day/night temperatures) were imbibed in water for 5 min at room temperature. Five seeds from each treatment were placed on a 1% water agar plates and allowed to germinate for 12 h in an incubator maintained at 30 °C. Following this, the seeds were dissected into several small pieces (2–4 mm cubes) from a defined area (Figure S2) with the help of a sharp razor. The seed tissue was immediately fixed in 2.5% glutaraldehyde buffered at 7.2 with 100 mM sodium cacodylate buffer for 4 h at room temperature. After several washes with water, the seed tissues were post fixed with 1% aqueous osmium tetroxide for 1 h. Excess osmium tetroxide was removed by washing the samples three times (15 min each) in water. The samples were dehydrated in a graded acetone series followed by gradual infiltration in Spurr’s resin. For each treatment, a minimum of 3 seed samples were examined under electron microscopy. Ultra-thin sections of embedded tissues were sectioned with a rotary microtome utilizing a diamond knife and collected on 200 mesh copper grids. The sections were double stained with lead citrate and uranyl acetate at room temperature. Stained sections examined in a JEOL 1200 EX (Tokyo, Japan) transmission electron microscope at 80 kV.

## Supplementary Materials

The following are available online at https://www.mdpi.com/1422-0067/21/13/4775/s1, Figure S1: Two-dimensional gel electrophoresis of seed proteins isolated from tolerant (TG) and heat stress sensitive (SG) genotypes grown at different growth temperatures. Figure S2: Longitudinal view of a paraffin embedded soybean seed section revealing the area (boxed) used for ultrastructure analysis.

## Abbreviations

HSP	Heat shock protein
PSV	Protein storage vacuoles
TG	Soybean genotype DS25-1, tolerant to elevated temperatures during seed fill
SG	Soybean genotype DT97-4290, sensitive to elevated temperatures during seed fill
SDS-PAGE	Sodium dodecyl sulfate–polyacrylamide gel electrophoresis
LOX	Lipoxygenase
KTI	Kunitz Trypsin inhibitor
BBI	Bowman-Birk inhibitor
