# Supplementary material for: Effect of Heat Stress on Seed Protein Composition and Ultrastructure of Protein Storage Vacuoles in the Cotyledonary Parenchyma Cells of Soybean Genotypes That Are Either Tolerant or Sensitive to Elevated Temperatures
Source: Int J Mol Sci. 2020 Jul 5;21(13):4775. doi: 10.3390/ijms21134775 (PMC7370294; doi:10.3390/ijms21134775)
Supplement: Supplementary file 1 [file ijms-21-04775-s001.pdf]

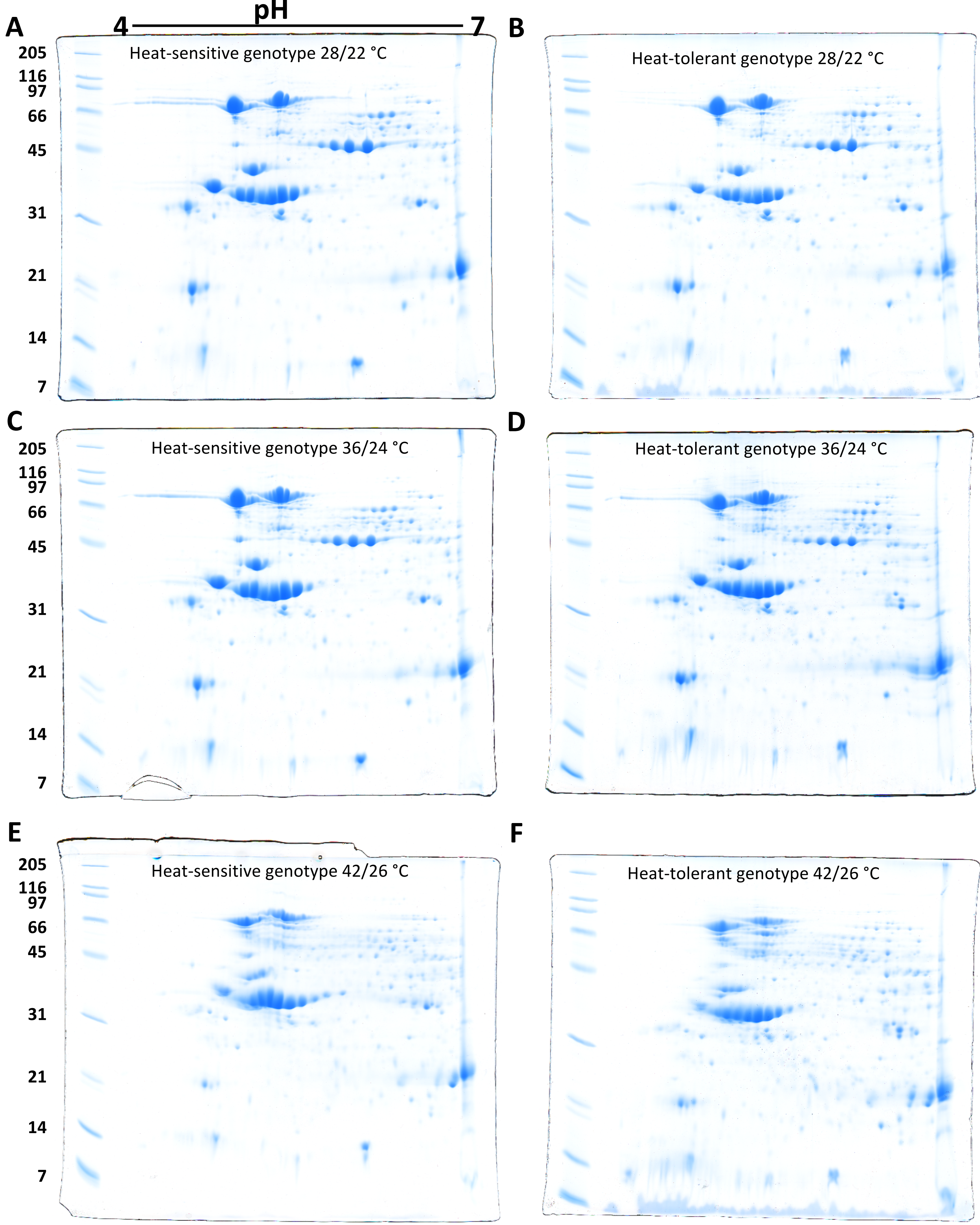

A
B
C
D
E
F
pH
4
7
Heat-sensitive genotype 28/22 °C
Heat-tolerant genotype 28/22 °C
Heat-sensitive genotype 36/24 °C
Heat-tolerant genotype 36/24 °C
Heat-sensitive genotype 42/26 °C
Heat-tolerant genotype 42/26 °C
205
116
97
66
45
31
21
14
7

Supplemental Figure 2

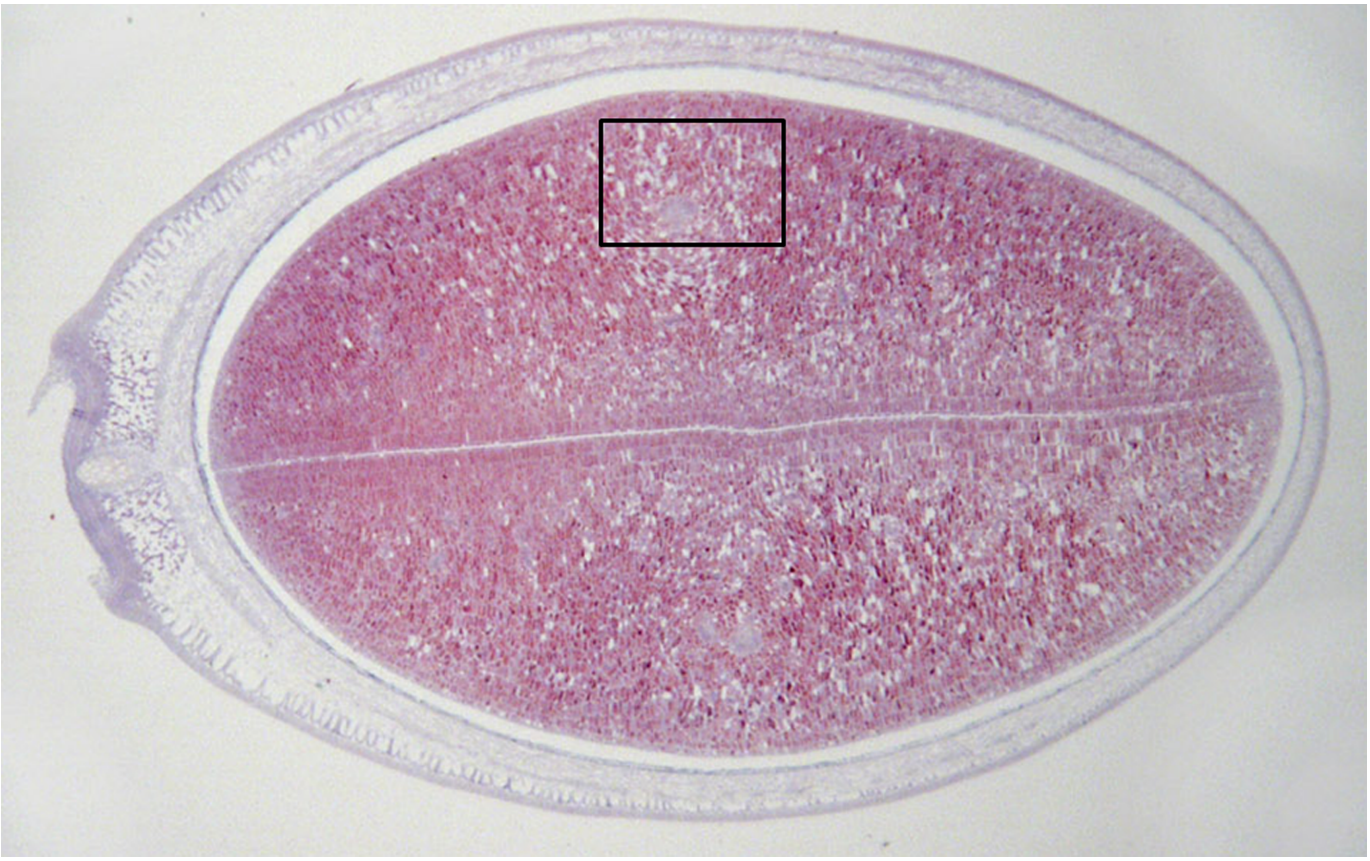

Longitudinal view of a paraffin embedded soybean seed section revealing the area (boxed) used for ultrastructure analysis.
